# *N*′-(3,5-Di-*tert*-butyl-4-hydroxy­benzyl­idene)-2-hydroxy­benzohydrazide methanol solvate

**DOI:** 10.1107/S1600536808020746

**Published:** 2008-07-09

**Authors:** Wagee A. Yehye, Azhar Ariffin, Seik Weng Ng

**Affiliations:** a Department of Chemistry, University of Malaya, 50603 Kuala Lumpur, Malaysia

## Abstract

The asymmetric unit of the title compound, C_22_H_28_N_2_O_3_·CH_4_O, consists of two independent Schiff base mol­ecules and two independent methanol solvent mol­ecules. In one Schiff base mol­ecule, the 2-hydr­oxy group forms an intra­molecular hydrogen bond with the amide O atom, whereas in the other Schiff base mol­ecule, the 2-hydr­oxy-substituted benzene ring is oriented so that the 2-hydr­oxy group serves as hydrogen-bond acceptor for the amide NH group. In the crystal structure, Schiff base mol­ecules inter­act with methanol solvent to furnish a hydrogen-bonded chain.

## Related literature

For references to other crystal structures of substituted benzyl­idene-2-hydroxy­benzohydrazides, see: Yehye *et al.* (2008 ▶).
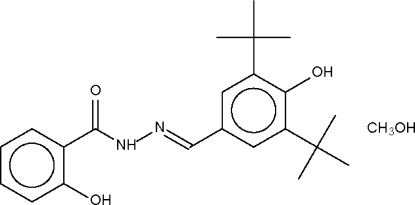


## Experimental

### 

#### Crystal data

C_22_H_28_N_2_O_3_·CH_4_O
*M* *_r_* = 400.51
Monoclinic, 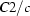

*a* = 24.184 (4) Å
*b* = 11.198 (2) Å
*c* = 33.112 (5) Å
β = 96.389 (3)°
*V* = 8911 (2) Å^3^
*Z* = 16
Mo *K*α radiation
μ = 0.08 mm^−1^
*T* = 100 (2) K
0.35 × 0.20 × 0.15 mm

#### Data collection

Bruker SMART APEX diffractometer
Absorption correction: none
22590 measured reflections
7825 independent reflections
4711 reflections with *I* > 2σ(*I*)
*R*_int_ = 0.090

#### Refinement

*R*[*F* ^2^ > 2σ(*F* ^2^)] = 0.074
*wR*(*F* ^2^) = 0.239
*S* = 1.09
7825 reflections
529 parameters
H-atom parameters constrained
Δρ_max_ = 0.74 e Å^−3^
Δρ_min_ = −0.58 e Å^−3^

### 

Data collection: *APEX2* (Bruker, 2007 ▶); cell refinement: *SAINT* (Bruker, 2007 ▶); data reduction: *SAINT*; program(s) used to solve structure: *SHELXS97* (Sheldrick, 2008 ▶); program(s) used to refine structure: *SHELXL97* (Sheldrick, 2008 ▶); molecular graphics: *X-SEED* (Barbour, 2001 ▶); software used to prepare material for publication: *publCIF* (Westrip, 2008 ▶).

## Supplementary Material

Crystal structure: contains datablocks global, I. DOI: 10.1107/S1600536808020746/lh2656sup1.cif

Structure factors: contains datablocks I. DOI: 10.1107/S1600536808020746/lh2656Isup2.hkl

Additional supplementary materials:  crystallographic information; 3D view; checkCIF report

## Figures and Tables

**Table 1:** Hydrogen-bond geometry (Å, °)

*D*—H⋯*A*	*D*—H	H⋯*A*	*D*⋯*A*	*D*—H⋯*A*
O1—H1*o*⋯O2	0.84	1.78	2.528 (4)	147
O4—H4*o*⋯O8	0.84	1.75	2.578 (4)	167
N1—H1*n*⋯O5^i^	0.88	2.10	2.763 (4)	132
N3—H3*n*⋯O4	0.88	1.88	2.592 (4)	137
O7—H7*o*⋯N2	0.84	2.16	2.900 (4)	148
O8—H8*o*⋯O7	0.84	2.00	2.704 (5)	140

Symmetry code: (i) 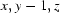.

**Fig. 1.**
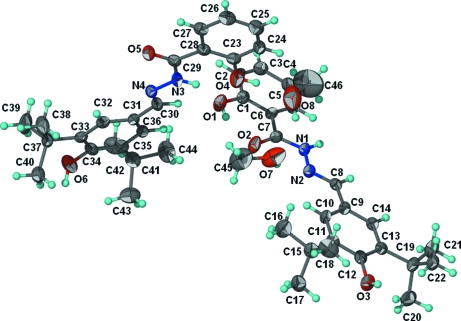
Thermal ellipsoid plot (Barbour, 2001) of C21H26N2O3.CH3OH at the 70% probability level. Hydrogen atoms are drawn as spheres of arbitrary radiius.

## supplementary crystallographic information

### Comment

The crystal structures of a number of substituted benzylidene-2-hydroxybenzohydrazides have been reported, along with that of the 2,4-dimethoxy derivative, which crystallizes as an ethanol solvate (Yehye *et al.*, 2008 and references cited within). In the title compound , the asymmetric unit consists of two Schiff-base and two solvent molecules. In one Schiff base molecule, the hydroxy group forms an intramolecular hydrogen bond with the amido C=O oxygen atom whereas in the other Schiff base molecule, the phenylene ring is rotated so that the 2-hydroxy group now serves as hydrogen-bond acceptor to the amido NH nitrogen atom (Fig. 1). The Schiff-base molecules interact with the two lattice methanol molecules to furnish a hydrogen-bonded chain.

### Experimental

2-Hydroxybenzohydrazide (0.5 g, 4 mmol) and 3,5-di-*tert*-butyl-4-hydroxybenzaldehyde (0.9 g, 4 mmol) were heated in ethanol (30 ml) for 2 h. The solvent was removed by evaporation and the product recrystallized from methanol.

### Refinement

Carbon-bound H-atoms were placed in calculated positions (C—H 0.95 to 0.98 Å) and were included in the refinement in the riding model approximation, with *U*(H) set to 1.2–1.5 *U*(C).

The oxygen- and nitrogen-bound H-atoms were similarly treated as riding (O–H 0.84 Å, N–H 0.88 Å).

### Crystal data

**Table:**

C_22_H_28_N_2_O_3_·CH_4_O	*F*_000_ = 3456
*M**_r_* = 400.51	*D*_x_ = 1.194 Mg m^−^^3^
Monoclinic, *C*2/*c*	Mo *K*α radiation λ = 0.71073 Å
Hall symbol: -C 2yc	Cell parameters from 2005 reflections
*a* = 24.184 (4) Å	θ = 2.3–19.8º
*b* = 11.198 (2) Å	µ = 0.08 mm^−^^1^
*c* = 33.112 (5) Å	*T* = 100 (2) K
β = 96.389 (3)º	Block, colorless
*V* = 8911 (2) Å^3^	0.35 × 0.20 × 0.15 mm
*Z* = 16	

### Data collection

**Table:**

Bruker SMART APEX diffractometer	4711 reflections with *I* > 2σ(*I*)
Radiation source: fine-focus sealed tube	*R*_int_ = 0.091
Monochromator: graphite	θ_max_ = 27.5º
*T* = 100(2) K	θ_min_ = 1.2º
ω scans	*h* = −29→31
Absorption correction: none	*k* = −14→14
22590 measured reflections	*l* = −37→43
7825 independent reflections	

### Refinement

**Table:**

Refinement on *F*^2^	Secondary atom site location: difference Fourier map
Least-squares matrix: full	Hydrogen site location: inferred from neighbouring sites
*R*[*F*^2^ > 2σ(*F*^2^)] = 0.074	H-atom parameters constrained
*wR*(*F*^2^) = 0.239	*w* = 1/[σ^2^(*F*_o_^2^) + (0.1232*P*)^2^] where *P* = (*F*_o_^2^ + 2*F*_c_^2^)/3
*S* = 1.09	(Δ/σ)_max_ = 0.001
7825 reflections	Δρ_max_ = 0.74 e Å^−^^3^
529 parameters	Δρ_min_ = −0.58 e Å^−^^3^
Primary atom site location: structure-invariant direct methods	Extinction correction: none

### Special details

**Table:**

Geometry. All e.s.d.'s (except the e.s.d. in the dihedral angle between two l.s. planes) are estimated using the full covariance matrix. The cell e.s.d.'s are taken into account individually in the estimation of e.s.d.'s in distances, angles and torsion angles; correlations between e.s.d.'s in cell parameters are only used when they are defined by crystal symmetry. An approximate (isotropic) treatment of cell e.s.d.'s is used for estimating e.s.d.'s involving l.s. planes.

### Fractional atomic coordinates and isotropic or equivalent isotropic displacement parameters (Å^2^)

**Table:**

	*x*	*y*	*z*	*U*_iso_*/*U*_eq_	
O1	0.41029 (12)	0.4254 (2)	0.44485 (9)	0.0356 (7)	
H1O	0.3871	0.4200	0.4241	0.053*	
O2	0.34515 (11)	0.3243 (2)	0.39032 (8)	0.0342 (7)	
O3	0.11317 (11)	0.0188 (2)	0.20608 (8)	0.0353 (7)	
H3O	0.0785	0.0210	0.2001	0.053*	
O4	0.22728 (12)	0.6204 (2)	0.44067 (10)	0.0429 (8)	
H4O	0.2122	0.5528	0.4390	0.064*	
O5	0.26877 (11)	0.9807 (2)	0.44825 (8)	0.0294 (6)	
O6	0.57906 (11)	0.8192 (2)	0.34770 (9)	0.0386 (7)	
H6O	0.5839	0.8150	0.3230	0.058*	
O7	0.23156 (15)	0.3840 (3)	0.35578 (11)	0.0607 (10)	
H7O	0.2558	0.3310	0.3540	0.091*	
O8	0.19213 (18)	0.4046 (3)	0.42874 (13)	0.0748 (12)	
H8O	0.1911	0.3752	0.4053	0.112*	
N1	0.31735 (12)	0.1345 (2)	0.39729 (9)	0.0227 (7)	
H1N	0.3206	0.0665	0.4107	0.027*	
N2	0.27940 (12)	0.1464 (3)	0.36298 (9)	0.0231 (7)	
N3	0.29514 (12)	0.7952 (3)	0.43174 (9)	0.0238 (7)	
H3N	0.2874	0.7184	0.4310	0.029*	
N4	0.34361 (12)	0.8351 (2)	0.41793 (9)	0.0225 (7)	
C1	0.41722 (15)	0.3175 (3)	0.46271 (12)	0.0259 (9)	
C2	0.45363 (16)	0.3079 (3)	0.49784 (12)	0.0307 (9)	
H2	0.4723	0.3770	0.5090	0.037*	
C3	0.46304 (15)	0.1999 (3)	0.51663 (12)	0.0304 (9)	
H3	0.4878	0.1948	0.5409	0.036*	
C4	0.43671 (16)	0.0973 (3)	0.50049 (11)	0.0294 (9)	
H4	0.4443	0.0222	0.5132	0.035*	
C5	0.39967 (15)	0.1052 (3)	0.46608 (11)	0.0269 (9)	
H5	0.3814	0.0353	0.4552	0.032*	
C6	0.38847 (15)	0.2155 (3)	0.44670 (11)	0.0212 (8)	
C7	0.34899 (15)	0.2288 (3)	0.40966 (11)	0.0244 (8)	
C8	0.25488 (15)	0.0499 (3)	0.35060 (11)	0.0219 (8)	
H8	0.2634	−0.0217	0.3654	0.026*	
C9	0.21440 (14)	0.0456 (3)	0.31464 (11)	0.0221 (8)	
C10	0.19724 (14)	0.1480 (3)	0.29246 (11)	0.0233 (8)	
H10	0.2097	0.2241	0.3024	0.028*	
C11	0.16278 (15)	0.1412 (3)	0.25655 (11)	0.0247 (8)	
C12	0.14477 (15)	0.0285 (3)	0.24271 (11)	0.0266 (9)	
C13	0.15856 (14)	−0.0763 (3)	0.26509 (11)	0.0231 (8)	
C14	0.19380 (14)	−0.0637 (3)	0.30063 (11)	0.0224 (8)	
H14	0.2043	−0.1331	0.3161	0.027*	
C15	0.14478 (16)	0.2559 (3)	0.23255 (12)	0.0304 (9)	
C16	0.16803 (18)	0.3676 (3)	0.25461 (13)	0.0392 (11)	
H16A	0.2087	0.3631	0.2585	0.059*	
H16B	0.1538	0.3731	0.2811	0.059*	
H16C	0.1565	0.4383	0.2384	0.059*	
C17	0.16714 (17)	0.2539 (4)	0.19088 (12)	0.0355 (10)	
H17A	0.2077	0.2460	0.1947	0.053*	
H17B	0.1569	0.3283	0.1764	0.053*	
H17C	0.1510	0.1860	0.1750	0.053*	
C18	0.08109 (17)	0.2675 (4)	0.22733 (13)	0.0438 (11)	
H18A	0.0674	0.2684	0.2541	0.066*	
H18B	0.0648	0.1996	0.2116	0.066*	
H18C	0.0704	0.3420	0.2130	0.066*	
C19	0.13496 (15)	−0.1998 (3)	0.25148 (12)	0.0264 (9)	
C20	0.15632 (18)	−0.2376 (4)	0.21133 (13)	0.0435 (11)	
H20A	0.1971	−0.2385	0.2148	0.065*	
H20B	0.1431	−0.1807	0.1899	0.065*	
H20C	0.1424	−0.3176	0.2038	0.065*	
C21	0.15297 (17)	−0.2956 (3)	0.28285 (13)	0.0360 (10)	
H21A	0.1937	−0.3001	0.2868	0.054*	
H21B	0.1377	−0.3729	0.2733	0.054*	
H21C	0.1391	−0.2754	0.3087	0.054*	
C22	0.07069 (15)	−0.1977 (3)	0.24675 (12)	0.0300 (9)	
H22A	0.0564	−0.2766	0.2382	0.045*	
H22B	0.0572	−0.1382	0.2263	0.045*	
H22C	0.0576	−0.1768	0.2728	0.045*	
C23	0.19310 (15)	0.6993 (3)	0.45657 (11)	0.0242 (8)	
C24	0.14403 (15)	0.6612 (3)	0.46956 (11)	0.0273 (9)	
H24	0.1345	0.5790	0.4677	0.033*	
C25	0.10856 (16)	0.7406 (4)	0.48518 (12)	0.0321 (9)	
H25	0.0743	0.7132	0.4933	0.038*	
C26	0.12249 (16)	0.8591 (4)	0.48915 (12)	0.0317 (9)	
H26	0.0982	0.9134	0.5004	0.038*	
C27	0.17232 (15)	0.8997 (3)	0.47672 (11)	0.0260 (9)	
H27	0.1820	0.9817	0.4797	0.031*	
C28	0.20813 (15)	0.8210 (3)	0.45986 (11)	0.0234 (8)	
C29	0.25946 (15)	0.8724 (3)	0.44643 (10)	0.0234 (8)	
C30	0.37778 (15)	0.7511 (3)	0.41175 (10)	0.0239 (8)	
H30	0.3683	0.6715	0.4182	0.029*	
C31	0.43033 (14)	0.7719 (3)	0.39532 (10)	0.0211 (8)	
C32	0.44696 (14)	0.8847 (3)	0.38361 (10)	0.0224 (8)	
H32	0.4234	0.9513	0.3866	0.027*	
C33	0.49662 (14)	0.9020 (3)	0.36783 (10)	0.0230 (8)	
C34	0.53000 (15)	0.8001 (3)	0.36417 (11)	0.0246 (8)	
C35	0.51542 (15)	0.6862 (3)	0.37589 (11)	0.0245 (8)	
C36	0.46473 (15)	0.6753 (3)	0.39136 (11)	0.0239 (8)	
H36	0.4533	0.5987	0.3995	0.029*	
C37	0.51376 (15)	1.0271 (3)	0.35460 (11)	0.0273 (9)	
C38	0.47007 (19)	1.1200 (3)	0.36147 (14)	0.0432 (12)	
H38A	0.4823	1.1985	0.3528	0.065*	
H38B	0.4649	1.1227	0.3904	0.065*	
H38C	0.4348	1.0989	0.3456	0.065*	
C39	0.56875 (19)	1.0644 (4)	0.37960 (15)	0.0524 (13)	
H39A	0.5796	1.1440	0.3711	0.079*	
H39B	0.5980	1.0069	0.3751	0.079*	
H39C	0.5636	1.0659	0.4085	0.079*	
C40	0.52080 (17)	1.0296 (3)	0.30912 (12)	0.0343 (10)	
H40A	0.5319	1.1100	0.3015	0.051*	
H40B	0.4855	1.0083	0.2933	0.051*	
H40C	0.5495	0.9722	0.3035	0.051*	
C41	0.55184 (16)	0.5756 (3)	0.37138 (12)	0.0329 (10)	
C42	0.60992 (17)	0.5924 (4)	0.39523 (13)	0.0435 (11)	
H42A	0.6059	0.6071	0.4239	0.065*	
H42B	0.6286	0.6607	0.3841	0.065*	
H42C	0.6322	0.5201	0.3928	0.065*	
C43	0.55643 (18)	0.5518 (4)	0.32594 (13)	0.0442 (11)	
H43A	0.5191	0.5404	0.3116	0.066*	
H43B	0.5787	0.4797	0.3232	0.066*	
H43C	0.5744	0.6201	0.3143	0.066*	
C44	0.52758 (19)	0.4641 (3)	0.38890 (16)	0.0502 (13)	
H44A	0.4905	0.4487	0.3747	0.075*	
H44B	0.5247	0.4761	0.4179	0.075*	
H44C	0.5519	0.3958	0.3853	0.075*	
C45	0.2538 (2)	0.4987 (4)	0.34603 (17)	0.0684 (16)	
H45A	0.2250	0.5599	0.3467	0.103*	
H45B	0.2660	0.4957	0.3188	0.103*	
H45C	0.2855	0.5184	0.3660	0.103*	
C46	0.2127 (4)	0.3167 (6)	0.4583 (2)	0.123 (3)	
H46A	0.2461	0.3474	0.4745	0.185*	
H46B	0.2219	0.2433	0.4444	0.185*	
H46C	0.1841	0.2995	0.4763	0.185*	

### Atomic displacement parameters (Å^2^)

**Table:**

	*U*^11^	*U*^22^	*U*^33^	*U*^12^	*U*^13^	*U*^23^
O1	0.0406 (18)	0.0229 (14)	0.0412 (18)	−0.0045 (12)	−0.0040 (13)	0.0005 (13)
O2	0.0429 (18)	0.0181 (14)	0.0379 (17)	−0.0037 (12)	−0.0116 (13)	0.0051 (12)
O3	0.0337 (17)	0.0385 (16)	0.0287 (16)	−0.0108 (12)	−0.0179 (12)	0.0070 (13)
O4	0.0356 (18)	0.0349 (16)	0.061 (2)	−0.0053 (13)	0.0162 (15)	−0.0083 (16)
O5	0.0300 (16)	0.0244 (14)	0.0346 (16)	−0.0022 (11)	0.0070 (12)	0.0019 (12)
O6	0.0275 (16)	0.0450 (17)	0.0456 (18)	0.0027 (13)	0.0147 (13)	0.0094 (14)
O7	0.077 (3)	0.0436 (19)	0.056 (2)	0.0226 (17)	−0.0180 (19)	−0.0070 (17)
O8	0.080 (3)	0.046 (2)	0.102 (3)	−0.0073 (19)	0.029 (3)	−0.001 (2)
N1	0.0246 (17)	0.0208 (16)	0.0208 (16)	−0.0034 (13)	−0.0066 (13)	0.0035 (13)
N2	0.0212 (17)	0.0249 (16)	0.0213 (16)	−0.0027 (13)	−0.0057 (13)	0.0034 (14)
N3	0.0200 (17)	0.0213 (16)	0.0303 (18)	−0.0023 (13)	0.0042 (14)	0.0023 (14)
N4	0.0185 (16)	0.0253 (16)	0.0238 (17)	−0.0022 (13)	0.0027 (13)	0.0013 (14)
C1	0.022 (2)	0.026 (2)	0.030 (2)	−0.0014 (16)	0.0063 (17)	−0.0043 (17)
C2	0.029 (2)	0.033 (2)	0.029 (2)	−0.0069 (18)	0.0001 (18)	−0.0107 (18)
C3	0.021 (2)	0.043 (2)	0.026 (2)	−0.0047 (18)	−0.0007 (17)	−0.0019 (19)
C4	0.029 (2)	0.032 (2)	0.026 (2)	−0.0021 (17)	−0.0017 (17)	0.0058 (18)
C5	0.027 (2)	0.0259 (19)	0.027 (2)	−0.0027 (16)	−0.0010 (17)	−0.0036 (17)
C6	0.022 (2)	0.0194 (18)	0.0213 (19)	0.0016 (15)	−0.0001 (15)	−0.0025 (15)
C7	0.027 (2)	0.023 (2)	0.023 (2)	−0.0003 (16)	0.0025 (16)	−0.0023 (17)
C8	0.024 (2)	0.0210 (19)	0.0204 (19)	0.0012 (16)	0.0023 (15)	0.0044 (16)
C9	0.020 (2)	0.0210 (19)	0.026 (2)	−0.0012 (15)	0.0026 (15)	−0.0006 (16)
C10	0.024 (2)	0.0194 (18)	0.026 (2)	−0.0029 (15)	−0.0011 (16)	−0.0004 (16)
C11	0.021 (2)	0.026 (2)	0.027 (2)	0.0027 (16)	0.0015 (16)	0.0069 (17)
C12	0.021 (2)	0.034 (2)	0.024 (2)	−0.0065 (16)	−0.0014 (16)	0.0038 (17)
C13	0.0173 (19)	0.030 (2)	0.022 (2)	−0.0030 (15)	0.0012 (15)	0.0012 (16)
C14	0.022 (2)	0.0202 (18)	0.026 (2)	0.0023 (15)	0.0040 (16)	0.0021 (16)
C15	0.030 (2)	0.031 (2)	0.029 (2)	0.0032 (17)	−0.0061 (17)	0.0083 (18)
C16	0.049 (3)	0.026 (2)	0.039 (3)	0.0062 (19)	−0.010 (2)	0.0105 (19)
C17	0.034 (2)	0.038 (2)	0.033 (2)	−0.0044 (19)	−0.0040 (19)	0.0092 (19)
C18	0.033 (3)	0.053 (3)	0.043 (3)	0.014 (2)	−0.003 (2)	0.014 (2)
C19	0.017 (2)	0.028 (2)	0.033 (2)	−0.0039 (16)	0.0008 (16)	−0.0062 (18)
C20	0.035 (3)	0.052 (3)	0.044 (3)	−0.015 (2)	0.010 (2)	−0.019 (2)
C21	0.033 (2)	0.025 (2)	0.048 (3)	0.0001 (18)	−0.005 (2)	−0.0034 (19)
C22	0.021 (2)	0.032 (2)	0.036 (2)	−0.0071 (17)	0.0010 (17)	−0.0039 (19)
C23	0.022 (2)	0.031 (2)	0.020 (2)	0.0029 (16)	0.0006 (16)	−0.0010 (16)
C24	0.023 (2)	0.033 (2)	0.026 (2)	−0.0068 (17)	0.0013 (17)	0.0020 (17)
C25	0.022 (2)	0.044 (2)	0.030 (2)	−0.0017 (18)	0.0005 (17)	0.0029 (19)
C26	0.023 (2)	0.042 (2)	0.030 (2)	0.0068 (18)	0.0046 (17)	−0.0001 (19)
C27	0.026 (2)	0.029 (2)	0.023 (2)	0.0053 (16)	0.0026 (16)	0.0045 (17)
C28	0.022 (2)	0.030 (2)	0.0177 (19)	0.0012 (16)	−0.0013 (15)	0.0031 (16)
C29	0.021 (2)	0.031 (2)	0.0180 (19)	0.0009 (16)	−0.0013 (15)	0.0035 (16)
C30	0.022 (2)	0.0259 (19)	0.024 (2)	−0.0046 (16)	0.0017 (16)	0.0044 (16)
C31	0.018 (2)	0.0246 (19)	0.0192 (19)	−0.0032 (15)	−0.0018 (15)	−0.0006 (16)
C32	0.021 (2)	0.0246 (19)	0.0201 (19)	0.0030 (15)	−0.0031 (15)	0.0002 (16)
C33	0.019 (2)	0.031 (2)	0.0181 (19)	−0.0037 (16)	−0.0034 (15)	−0.0027 (16)
C34	0.019 (2)	0.034 (2)	0.020 (2)	−0.0005 (16)	0.0015 (15)	0.0047 (17)
C35	0.024 (2)	0.027 (2)	0.022 (2)	0.0036 (16)	−0.0019 (16)	0.0047 (16)
C36	0.027 (2)	0.0233 (19)	0.020 (2)	−0.0038 (16)	−0.0003 (16)	0.0019 (16)
C37	0.026 (2)	0.027 (2)	0.028 (2)	−0.0081 (16)	−0.0010 (16)	0.0027 (17)
C38	0.052 (3)	0.023 (2)	0.058 (3)	−0.0012 (19)	0.020 (2)	0.006 (2)
C39	0.048 (3)	0.052 (3)	0.053 (3)	−0.029 (2)	−0.015 (2)	0.008 (2)
C40	0.038 (3)	0.029 (2)	0.036 (2)	−0.0051 (18)	0.0030 (19)	0.0038 (19)
C41	0.030 (2)	0.030 (2)	0.039 (2)	0.0071 (17)	0.0091 (19)	0.0029 (19)
C42	0.039 (3)	0.052 (3)	0.039 (3)	0.018 (2)	0.000 (2)	0.010 (2)
C43	0.039 (3)	0.052 (3)	0.042 (3)	0.013 (2)	0.008 (2)	−0.009 (2)
C44	0.050 (3)	0.027 (2)	0.077 (4)	0.014 (2)	0.026 (3)	0.004 (2)
C45	0.097 (5)	0.037 (3)	0.066 (4)	0.020 (3)	−0.012 (3)	0.007 (3)
C46	0.203 (9)	0.065 (4)	0.103 (6)	0.038 (5)	0.022 (6)	0.030 (4)

### Geometric parameters (Å, °)

**Table:**

O1—C1	1.348 (4)	C20—H20B	0.9800
O1—H1O	0.8400	C20—H20C	0.9800
O2—C7	1.245 (4)	C21—H21A	0.9800
O3—C12	1.364 (4)	C21—H21B	0.9800
O3—H3O	0.8402	C21—H21C	0.9800
O4—C23	1.356 (4)	C22—H22A	0.9800
O4—H4O	0.8400	C22—H22B	0.9800
O5—C29	1.233 (4)	C22—H22C	0.9800
O6—C34	1.377 (4)	C23—C24	1.374 (5)
O6—H6O	0.8400	C23—C28	1.411 (5)
O7—C45	1.443 (6)	C24—C25	1.376 (5)
O7—H7O	0.8400	C24—H24	0.9500
O8—C46	1.438 (7)	C25—C26	1.371 (6)
O8—H8O	0.8400	C25—H25	0.9500
N1—C7	1.341 (4)	C26—C27	1.393 (5)
N1—N2	1.385 (4)	C26—H26	0.9500
N1—H1N	0.8800	C27—C28	1.395 (5)
N2—C8	1.278 (4)	C27—H27	0.9500
N3—C29	1.350 (4)	C28—C29	1.481 (5)
N3—N4	1.379 (4)	C30—C31	1.456 (5)
N3—H3N	0.8800	C30—H30	0.9500
N4—C30	1.284 (4)	C31—C36	1.380 (5)
C1—C2	1.383 (5)	C31—C32	1.393 (5)
C1—C6	1.409 (5)	C32—C33	1.376 (5)
C2—C3	1.368 (5)	C32—H32	0.9500
C2—H2	0.9500	C33—C34	1.410 (5)
C3—C4	1.391 (5)	C33—C37	1.539 (5)
C3—H3	0.9500	C34—C35	1.390 (5)
C4—C5	1.371 (5)	C35—C36	1.386 (5)
C4—H4	0.9500	C35—C41	1.537 (5)
C5—C6	1.404 (5)	C36—H36	0.9500
C5—H5	0.9500	C37—C38	1.518 (5)
C6—C7	1.476 (5)	C37—C40	1.534 (5)
C8—C9	1.456 (5)	C37—C39	1.544 (5)
C8—H8	0.9500	C38—H38A	0.9800
C9—C14	1.382 (5)	C38—H38B	0.9800
C9—C10	1.400 (5)	C38—H38C	0.9800
C10—C11	1.376 (5)	C39—H39A	0.9800
C10—H10	0.9500	C39—H39B	0.9800
C11—C12	1.396 (5)	C39—H39C	0.9800
C11—C15	1.547 (5)	C40—H40A	0.9800
C12—C13	1.409 (5)	C40—H40B	0.9800
C13—C14	1.381 (5)	C40—H40C	0.9800
C13—C19	1.544 (5)	C41—C44	1.521 (5)
C14—H14	0.9500	C41—C43	1.544 (6)
C15—C16	1.523 (5)	C41—C42	1.544 (6)
C15—C18	1.536 (5)	C42—H42A	0.9800
C15—C17	1.537 (5)	C42—H42B	0.9800
C16—H16A	0.9800	C42—H42C	0.9800
C16—H16B	0.9800	C43—H43A	0.9800
C16—H16C	0.9800	C43—H43B	0.9800
C17—H17A	0.9800	C43—H43C	0.9800
C17—H17B	0.9800	C44—H44A	0.9800
C17—H17C	0.9800	C44—H44B	0.9800
C18—H18A	0.9800	C44—H44C	0.9800
C18—H18B	0.9800	C45—H45A	0.9800
C18—H18C	0.9800	C45—H45B	0.9800
C19—C21	1.522 (5)	C45—H45C	0.9800
C19—C20	1.538 (5)	C46—H46A	0.9800
C19—C22	1.545 (5)	C46—H46B	0.9800
C20—H20A	0.9800	C46—H46C	0.9800
			
C1—O1—H1O	109.5	H22B—C22—H22C	109.5
C12—O3—H3O	130.8	O4—C23—C24	120.3 (3)
C23—O4—H4O	109.5	O4—C23—C28	119.7 (3)
C34—O6—H6O	126.7	C24—C23—C28	120.0 (3)
C45—O7—H7O	109.5	C23—C24—C25	120.9 (4)
C46—O8—H8O	109.5	C23—C24—H24	119.6
C7—N1—N2	118.5 (3)	C25—C24—H24	119.6
C7—N1—H1N	120.7	C26—C25—C24	120.3 (4)
N2—N1—H1N	120.7	C26—C25—H25	119.9
C8—N2—N1	115.0 (3)	C24—C25—H25	119.9
C29—N3—N4	120.9 (3)	C25—C26—C27	119.9 (4)
C29—N3—H3N	119.6	C25—C26—H26	120.0
N4—N3—H3N	119.6	C27—C26—H26	120.0
C30—N4—N3	113.7 (3)	C26—C27—C28	120.5 (4)
O1—C1—C2	118.4 (3)	C26—C27—H27	119.7
O1—C1—C6	122.0 (3)	C28—C27—H27	119.7
C2—C1—C6	119.7 (3)	C27—C28—C23	118.4 (3)
C3—C2—C1	120.6 (4)	C27—C28—C29	116.9 (3)
C3—C2—H2	119.7	C23—C28—C29	124.7 (3)
C1—C2—H2	119.7	O5—C29—N3	121.8 (3)
C2—C3—C4	120.6 (4)	O5—C29—C28	121.5 (3)
C2—C3—H3	119.7	N3—C29—C28	116.7 (3)
C4—C3—H3	119.7	N4—C30—C31	123.1 (3)
C5—C4—C3	119.7 (4)	N4—C30—H30	118.4
C5—C4—H4	120.2	C31—C30—H30	118.4
C3—C4—H4	120.2	C36—C31—C32	119.2 (3)
C4—C5—C6	120.8 (3)	C36—C31—C30	118.1 (3)
C4—C5—H5	119.6	C32—C31—C30	122.7 (3)
C6—C5—H5	119.6	C33—C32—C31	121.6 (3)
C5—C6—C1	118.6 (3)	C33—C32—H32	119.2
C5—C6—C7	122.9 (3)	C31—C32—H32	119.2
C1—C6—C7	118.5 (3)	C32—C33—C34	116.8 (3)
O2—C7—N1	120.8 (3)	C32—C33—C37	120.7 (3)
O2—C7—C6	121.2 (3)	C34—C33—C37	122.5 (3)
N1—C7—C6	118.0 (3)	O6—C34—C35	120.7 (3)
N2—C8—C9	122.4 (3)	O6—C34—C33	115.8 (3)
N2—C8—H8	118.8	C35—C34—C33	123.5 (3)
C9—C8—H8	118.8	C36—C35—C34	116.5 (3)
C14—C9—C10	118.4 (3)	C36—C35—C41	120.4 (3)
C14—C9—C8	119.2 (3)	C34—C35—C41	123.1 (3)
C10—C9—C8	122.4 (3)	C31—C36—C35	122.3 (3)
C11—C10—C9	121.6 (3)	C31—C36—H36	118.9
C11—C10—H10	119.2	C35—C36—H36	118.9
C9—C10—H10	119.2	C38—C37—C40	106.8 (3)
C10—C11—C12	118.1 (3)	C38—C37—C33	111.6 (3)
C10—C11—C15	120.4 (3)	C40—C37—C33	111.0 (3)
C12—C11—C15	121.5 (3)	C38—C37—C39	107.9 (4)
O3—C12—C11	119.2 (3)	C40—C37—C39	109.8 (3)
O3—C12—C13	118.5 (3)	C33—C37—C39	109.7 (3)
C11—C12—C13	122.3 (3)	C37—C38—H38A	109.5
C14—C13—C12	116.8 (3)	C37—C38—H38B	109.5
C14—C13—C19	120.9 (3)	H38A—C38—H38B	109.5
C12—C13—C19	122.3 (3)	C37—C38—H38C	109.5
C13—C14—C9	122.8 (3)	H38A—C38—H38C	109.5
C13—C14—H14	118.6	H38B—C38—H38C	109.5
C9—C14—H14	118.6	C37—C39—H39A	109.5
C16—C15—C18	107.3 (3)	C37—C39—H39B	109.5
C16—C15—C17	107.2 (3)	H39A—C39—H39B	109.5
C18—C15—C17	110.4 (3)	C37—C39—H39C	109.5
C16—C15—C11	111.5 (3)	H39A—C39—H39C	109.5
C18—C15—C11	110.4 (3)	H39B—C39—H39C	109.5
C17—C15—C11	110.0 (3)	C37—C40—H40A	109.5
C15—C16—H16A	109.5	C37—C40—H40B	109.5
C15—C16—H16B	109.5	H40A—C40—H40B	109.5
H16A—C16—H16B	109.5	C37—C40—H40C	109.5
C15—C16—H16C	109.5	H40A—C40—H40C	109.5
H16A—C16—H16C	109.5	H40B—C40—H40C	109.5
H16B—C16—H16C	109.5	C44—C41—C35	112.0 (3)
C15—C17—H17A	109.5	C44—C41—C43	107.6 (4)
C15—C17—H17B	109.5	C35—C41—C43	109.8 (3)
H17A—C17—H17B	109.5	C44—C41—C42	105.8 (4)
C15—C17—H17C	109.5	C35—C41—C42	110.5 (3)
H17A—C17—H17C	109.5	C43—C41—C42	111.1 (3)
H17B—C17—H17C	109.5	C41—C42—H42A	109.5
C15—C18—H18A	109.5	C41—C42—H42B	109.5
C15—C18—H18B	109.5	H42A—C42—H42B	109.5
H18A—C18—H18B	109.5	C41—C42—H42C	109.5
C15—C18—H18C	109.5	H42A—C42—H42C	109.5
H18A—C18—H18C	109.5	H42B—C42—H42C	109.5
H18B—C18—H18C	109.5	C41—C43—H43A	109.5
C21—C19—C20	107.3 (3)	C41—C43—H43B	109.5
C21—C19—C13	111.3 (3)	H43A—C43—H43B	109.5
C20—C19—C13	110.5 (3)	C41—C43—H43C	109.5
C21—C19—C22	106.8 (3)	H43A—C43—H43C	109.5
C20—C19—C22	110.3 (3)	H43B—C43—H43C	109.5
C13—C19—C22	110.5 (3)	C41—C44—H44A	109.5
C19—C20—H20A	109.5	C41—C44—H44B	109.5
C19—C20—H20B	109.5	H44A—C44—H44B	109.5
H20A—C20—H20B	109.5	C41—C44—H44C	109.5
C19—C20—H20C	109.5	H44A—C44—H44C	109.5
H20A—C20—H20C	109.5	H44B—C44—H44C	109.5
H20B—C20—H20C	109.5	O7—C45—H45A	109.5
C19—C21—H21A	109.5	O7—C45—H45B	109.5
C19—C21—H21B	109.5	H45A—C45—H45B	109.5
H21A—C21—H21B	109.5	O7—C45—H45C	109.5
C19—C21—H21C	109.5	H45A—C45—H45C	109.5
H21A—C21—H21C	109.5	H45B—C45—H45C	109.5
H21B—C21—H21C	109.5	O8—C46—H46A	109.5
C19—C22—H22A	109.5	O8—C46—H46B	109.5
C19—C22—H22B	109.5	H46A—C46—H46B	109.5
H22A—C22—H22B	109.5	O8—C46—H46C	109.5
C19—C22—H22C	109.5	H46A—C46—H46C	109.5
H22A—C22—H22C	109.5	H46B—C46—H46C	109.5
			
C7—N1—N2—C8	173.1 (3)	C12—C13—C19—C22	−57.0 (4)
C29—N3—N4—C30	168.2 (3)	O4—C23—C24—C25	−179.0 (4)
O1—C1—C2—C3	−178.6 (3)	C28—C23—C24—C25	1.2 (6)
C6—C1—C2—C3	1.6 (6)	C23—C24—C25—C26	−1.9 (6)
C1—C2—C3—C4	0.8 (6)	C24—C25—C26—C27	1.1 (6)
C2—C3—C4—C5	−2.0 (6)	C25—C26—C27—C28	0.6 (6)
C3—C4—C5—C6	0.7 (6)	C26—C27—C28—C23	−1.3 (5)
C4—C5—C6—C1	1.7 (5)	C26—C27—C28—C29	178.4 (3)
C4—C5—C6—C7	−180.0 (3)	O4—C23—C28—C27	−179.4 (3)
O1—C1—C6—C5	177.4 (3)	C24—C23—C28—C27	0.4 (5)
C2—C1—C6—C5	−2.8 (5)	O4—C23—C28—C29	1.0 (6)
O1—C1—C6—C7	−1.1 (5)	C24—C23—C28—C29	−179.2 (3)
C2—C1—C6—C7	178.7 (3)	N4—N3—C29—O5	−0.3 (5)
N2—N1—C7—O2	−1.4 (5)	N4—N3—C29—C28	179.0 (3)
N2—N1—C7—C6	178.9 (3)	C27—C28—C29—O5	−3.4 (5)
C5—C6—C7—O2	−170.8 (3)	C23—C28—C29—O5	176.2 (3)
C1—C6—C7—O2	7.5 (5)	C27—C28—C29—N3	177.3 (3)
C5—C6—C7—N1	9.0 (5)	C23—C28—C29—N3	−3.1 (5)
C1—C6—C7—N1	−172.7 (3)	N3—N4—C30—C31	176.9 (3)
N1—N2—C8—C9	−179.0 (3)	N4—C30—C31—C36	177.6 (3)
N2—C8—C9—C14	173.4 (3)	N4—C30—C31—C32	−2.3 (6)
N2—C8—C9—C10	−3.7 (5)	C36—C31—C32—C33	0.6 (5)
C14—C9—C10—C11	−3.0 (5)	C30—C31—C32—C33	−179.4 (3)
C8—C9—C10—C11	174.1 (3)	C31—C32—C33—C34	−0.2 (5)
C9—C10—C11—C12	0.5 (5)	C31—C32—C33—C37	179.2 (3)
C9—C10—C11—C15	−179.6 (3)	C32—C33—C34—O6	179.0 (3)
C10—C11—C12—O3	−176.3 (3)	C37—C33—C34—O6	−0.4 (5)
C15—C11—C12—O3	3.8 (5)	C32—C33—C34—C35	−0.6 (5)
C10—C11—C12—C13	3.0 (5)	C37—C33—C34—C35	180.0 (3)
C15—C11—C12—C13	−176.9 (3)	O6—C34—C35—C36	−178.7 (3)
O3—C12—C13—C14	175.6 (3)	C33—C34—C35—C36	0.9 (6)
C11—C12—C13—C14	−3.7 (5)	O6—C34—C35—C41	−0.2 (6)
O3—C12—C13—C19	−5.6 (5)	C33—C34—C35—C41	179.4 (3)
C11—C12—C13—C19	175.1 (3)	C32—C31—C36—C35	−0.3 (5)
C12—C13—C14—C9	1.1 (5)	C30—C31—C36—C35	179.7 (3)
C19—C13—C14—C9	−177.8 (3)	C34—C35—C36—C31	−0.4 (5)
C10—C9—C14—C13	2.2 (5)	C41—C35—C36—C31	−178.9 (3)
C8—C9—C14—C13	−175.0 (3)	C32—C33—C37—C38	0.1 (5)
C10—C11—C15—C16	−2.1 (5)	C34—C33—C37—C38	179.4 (3)
C12—C11—C15—C16	177.9 (3)	C32—C33—C37—C40	−119.0 (4)
C10—C11—C15—C18	−121.2 (4)	C34—C33—C37—C40	60.4 (5)
C12—C11—C15—C18	58.7 (5)	C32—C33—C37—C39	119.5 (4)
C10—C11—C15—C17	116.7 (4)	C34—C33—C37—C39	−61.1 (5)
C12—C11—C15—C17	−63.4 (4)	C36—C35—C41—C44	−4.9 (5)
C14—C13—C19—C21	3.3 (5)	C34—C35—C41—C44	176.6 (4)
C12—C13—C19—C21	−175.5 (3)	C36—C35—C41—C43	114.5 (4)
C14—C13—C19—C20	−115.8 (4)	C34—C35—C41—C43	−63.9 (5)
C12—C13—C19—C20	65.4 (4)	C36—C35—C41—C42	−122.7 (4)
C14—C13—C19—C22	121.8 (4)	C34—C35—C41—C42	58.9 (5)

### Hydrogen-bond geometry (Å, °)

**Table:**

*D*—H···*A*	*D*—H	H···*A*	*D*···*A*	*D*—H···*A*
O1—H1o···O2	0.84	1.78	2.528 (4)	147
O4—H4o···O8	0.84	1.75	2.578 (4)	167
N1—H1n···O5^i^	0.88	2.10	2.763 (4)	132
N3—H3n···O4	0.88	1.88	2.592 (4)	137
O7—H7o···N2	0.84	2.16	2.900 (4)	148
O8—H8o···O7	0.84	2.00	2.704 (5)	140

Symmetry codes: (i) *x*, *y*−1, *z*.
